# Dr. Henry Lapp

**Published:** 1912-04

**Authors:** 


Dr. Henry Lapp of Clarence, a graduate of University of Buffalo, 1865, died March 1, 1912, aged 76. He was born at Clarence, educated at the Williamsville Academy, and spent 48 years in the active practice of his profession. He was a member of the county and state societies, of the Gross Medical Club, was formerly a Censor of the Medical Department of the University of Buffalo, President of the Medical Society of the County of Erie, and was widely and affectionately known in his profession. Beside a widow, two brothers and a son in business life, he is survived by a son, Dr. Henry C. Lapp, who is practicing medicine at Clarence.

